# The endogenous and reactive depression subtypes revisited: integrative animal and human studies implicate multiple distinct molecular mechanisms underlying major depressive disorder

**DOI:** 10.1186/1741-7015-12-73

**Published:** 2014-05-07

**Authors:** Karim Malki, Robert Keers, Maria Grazia Tosto, Anbarasu Lourdusamy, Lucia Carboni, Enrico Domenici, Rudolf Uher, Peter McGuffin, Leonard C Schalkwyk

**Affiliations:** 1 King’s College London, MRC Social, Genetic and Developmental Psychiatry Centre, at Institute of Psychiatry, SGDP Research Centre (PO80), De Crespigny Park, Denmark Hill, London SE5 8AF, UK; 2 Department of Psychology, University of York, York, UK; 3 Queen’s Medical Centre, University of Nottingham, Nottingham, UK; 4 Department of Pharmacy and Biotechnology, Alma Mater Studiorum, University of Bologna, Bologna, Italy; 5 Center of Excellence for Drug Discovery in Neuroscience, GlaxoSmithKline Medicines Research Centre, Verona, Italy; 6 Current address: Pharma Research and Early Development, F. Hoffmann–La Roche, Basel, Switzerland; 7 Department of Psychiatry, Dalhousie University, Halifax, NS, Canada

**Keywords:** Endogenous Depression, Reactive Depression, GENDEP, VAMP-2, DSM-IV, Stanley Brain Consortium, mRNA, Stress

## Abstract

**Background:**

Traditional diagnoses of major depressive disorder (MDD) suggested that the presence or absence of stress prior to onset results in either ‘reactive’ or ‘endogenous’ subtypes of the disorder, respectively. Several lines of research suggest that the biological underpinnings of ‘reactive’ or ‘endogenous’ subtypes may also differ, resulting in differential response to treatment. We investigated this hypothesis by comparing the gene-expression profiles of three animal models of ‘reactive’ and ‘endogenous’ depression. We then translated these findings to clinical samples using a human post-mortem mRNA study.

**Methods:**

Affymetrix mouse whole-genome oligonucleotide arrays were used to measure gene expression from hippocampal tissues of 144 mice from the Genome-based Therapeutic Drugs for Depression (GENDEP) project. The study used four inbred mouse strains and two depressogenic ‘stress’ protocols (maternal separation and Unpredictable Chronic Mild Stress) to model ‘reactive’ depression. Stress-related mRNA differences in mouse were compared with a parallel mRNA study using Flinders Sensitive and Resistant rat lines as a model of ‘endogenous’ depression. Convergent genes differentially expressed across the animal studies were used to inform candidate gene selection in a human mRNA post-mortem case control study from the Stanley Brain Consortium.

**Results:**

In the mouse ‘reactive’ model, the expression of 350 genes changed in response to early stresses and 370 in response to late stresses. A minimal genetic overlap (less than 8.8%) was detected in response to both stress protocols, but 30% of these genes (21) were also differentially regulated in the ‘endogenous’ rat study. This overlap is significantly greater than expected by chance. The *VAMP-2* gene, differentially expressed across the rodent studies, was also significantly altered in the human study after correcting for multiple testing.

**Conclusions:**

Our results suggest that ‘endogenous’ and ‘reactive’ subtypes of depression are associated with largely distinct changes in gene-expression. However, they also suggest that the molecular signature of ‘reactive’ depression caused by early stressors differs considerably from that of ‘reactive’ depression caused by late stressors. A small set of genes was consistently dysregulated across each paradigm and in post-mortem brain tissue of depressed patients suggesting a final common pathway to the disorder. These genes included the *VAMP-2* gene, which has previously been associated with Axis-I disorders including MDD, bipolar depression, schizophrenia and with antidepressant treatment response. We also discuss the implications of our findings for disease classification, personalized medicine and case-control studies of MDD.

## Background

Although antidepressants remain the first line treatment for major depressive disorder (MDD), antidepressant response varies considerably between individuals: fewer than half of all patients achieve remission following their first course of treatment [1]. The absence of robust predictors of treatment response means that the most effective antidepressant for a given patient is currently identified by trial and error. This is often a long and costly process which both delays recovery and has a negative effect on long-term outcome [2].

Clinicians have long intuited that heterogeneity in treatment response is the direct result of etiological heterogeneity in MDD [3]. Indeed, traditional diagnoses of major depression proposed that the presence or absence of stress prior to the onset of MDD results in two etiologically distinct subgroups of the disorder with different treatment recommendations. Early studies, which categorized these subtypes as ‘reactive’ (occurring as the result of a stressor) or ‘endogenous’ (occurring in the absence of stress), suggested that those with ‘endogenous’ depression responded more favorably to tricyclic antidepressants (TCAs) than selective serotonin reuptake inhibitors (SSRIs) [4]. While the validity of these subtypes remains unclear, reports continue to show that both distal stress (occurring early in life [5]) and proximal stress (occurring near the onset of a depressive episode [6]) are predictive of treatment response.

It remains unclear how the presence or absence of stress in the etiology of MDD affects response to treatment. However, it has been suggested that ‘endogenous’ and ‘reactive’ subtypes of depression are associated with largely distinct biological mechanisms, which respond differentially to treatment [3]. In line with this hypothesis, a recent animal study reported that the hippocampal gene-expression profile of a ‘reactive’ model of depression (induced by chronic restraint stress) differed considerably from that of an ‘endogenous’ model [7].

While this study suggests that the gene-expression profiles of ‘reactive’ depression caused by proximal stress may indeed differ from ‘endogenous’ depression, the role of distal early-life stress in this distinction remains unknown. Several studies have highlighted the importance of the timing of adversity and show that early and late stressors may have differential tissue-specific effects on gene-expression in the hippocampus [8-12]. The pathophysiological processes underlying MDD may therefore differ not only in the presence or absence of a stressor, but also by the timing of adversity (distal *vs*. proximal stress).

We investigated this hypothesis by exploring hippocampal gene-expression (mRNA) differences in three animal models of depression chosen to represent ‘reactive’ and ‘endogenous’ depression. In the ‘reactive’ depression model, mice were exposed to either distal stress (maternal separation) or proximal stress (unpredictable chronic mild stress). Flinders sensitive rats, which show congenital depression-like behavior, were used to model ‘endogenous’ depression.

Whole genome transcription profiles from disease relevant brain tissues in animals may provide valuable support and important information on the molecular mechanisms that may be relevant in humans. Nevertheless, the specific features of psychiatric illnesses means that molecular mechanisms uncovered in animal models are only suggestive and need to be validated in human studies [13,14]. We therefore used findings from the animal models to inform probe set prioritization in a comparable human post-mortem case-control study of depression from the Stanley Brain Consortium. Specifically, we hypothesize that a set of genes that shows concordant expression differences in response to ‘reactive’ and ‘endogenous’ depression models in the rodent studies may represent a common final pathway to MDD. These same genes may therefore also be differentially regulated in the post-mortem brain tissue of humans with the disorder.

## Methods

### Design

Genome-wide expression profiling of the hippocampus (HIP) from two studies from the rodent arm of the Genome-based Therapeutic Drugs for Depression (GENDEP) study [15] was used to inform candidate gene selection in a comparable human post-mortem, case-control study on MDD from the Stanley Brain Consortium. The GENDEP project is a large-scale, multi-center human pharmacogenomics study that also includes a series of large-scale studies using animal models and *in vitro* experiments. The GENDEP project was designed to allow for integrative analysis of the results of the transcriptomics and proteomics on the samples from the human, the rodent and the *in vitro* studies, in order to gain further insight into the molecular mechanisms of MDD and identify biomarkers of antidepressant drugs (AD) treatment response. The mouse study used 144 animals from four strains of well-characterized inbred mice to model individual variation in humans. The mice were subjected to one of two stress protocols and a control condition (maternal separation (MS) - ‘early stress’, unpredictable chronic mild stress (UCMS) - ‘late stress’ - or the control condition (ENV)) to model ‘reactive’ depression. Litters of each strain were randomly allocated to the MS, UCMS or control group. Findings from the mouse study were cross validated in a parallel rat study that compared HIP mRNA differences between Flinders Sensitive and Flinders Resistant rat lines as models of ‘endogenous’ depression. Finally, genes differentially expressed in response to both stress protocols in the mouse study and in the rat study were used to inform probe set selection in comparable mRNA expression study in humans.

### Animals

A total of 144 male and female mice (72 of each sex) from four different strains ((129S1/SvImJ, C57LB/6 J, DBA/2 J and FVB/NJ) were bred in the barrier unit at the Institute of Psychiatry, London, UK. Weaning took place when the animals were 21 to 28 days old. Animals were group-housed under standard conditions with a 12:12 h light:dark cycle, 22°C ± 11°C, food and water *ad libitum*. A total of 144 animals were sacrificed by cervical dislocation. Animals used for the transcriptomic study were not behaviorally tested. The hippocampus, liver and spleen were dissected following previously published protocols [16,17]. All housing and experimental procedures were carried out in accordance with the UK Home Office Animals (Scientific Procedures) Act, 1986.

A total of 39 animals from two cohorts of Flinders Sensitive Lines and Flinders Resistant Lines (22 FRL and 17 FSL) were bred and maintained at Karolinska Institutet (Stockholm) and housed under standard room temperature (22 ± 1°C), relative humidity (45 to 55%) and a 12 h light:dark schedule (light on at 07:00 a.m.). Food and water were available *ad libitum*. The study was conducted as part of a parallel GENDEP investigation. The Stockholm's Ethical Committee for Protection of Animals approved the study and all procedures were conducted in conformity with the Karolinska Institutet's guidelines for the care and use of laboratory animals, which follows the European Communities Council Directive of 24 November 1986. Additional information on the rat study is available elsewhere [18].

#### UCMS (Unpredictable Chronic Mild Stress)

**Figure 1:**
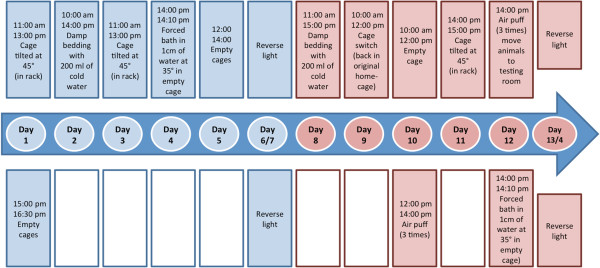
**This figure shows the stress administration regime for the unpredictable chronic mild stress paradigm.** The duration of the stress regime was for two consecutive weeks and the order of the different stressors was randomized. This figure shows the stressors and time/duration of administration for each of the two weeks.

In mice, ‘reactive’ depression caused by proximal stress was modeled using an Unpredictable Chronic Mild Stress (UCMS) paradigm. A third of the 144 mice (48 male and female mice) were exposed to varying stressors on a daily basis for a period of two weeks. Exposure to UCMS commenced when the animals were 10 weeks of age. The UCSM protocols included exposure to different stressors each day in a pseudorandom order. The stressors in the UCMS regime were based on previously published protocols including two hours of home cage tilting at 45°, damp bedding for four hours, cage switching for two hours, flooded cage for 10 minutes, altered length and time of light-dark cycle and air-puff [19]. Animals were exposed to either one or two stressors each day for varying lengths of time (Figure 1). All UCMS-exposed mice were tested and maintained under standard laboratory conditions but were single-housed. Following the UCMS regimen, a set of animals was tested with a battery of behavioral tests including Porsolt as an index of UCMS-evoked depressive-behavior [19]. However, all animals used for this mRNA characterization were not behaviorally tested to control for the potential stressor effects of the tests.

#### MS (Maternal Separation)

A maternal separation protocol was used to model ‘reactive’ depression caused by distal stress in a further 48 mice. A single 24-hour separation of the pup from the dam at postnatal day (PND) 9 protocol was chosen to elicit a sufficiently strong biological response. Day of birth was defined as PND 0 for that particular litter. On postnatal Day 9 the dam was removed from the litter for 24 hours. The litter was kept on a heating pad in their home cage at 33°C ± 2°C in a different room than the dam in order to avoid contact through vocalization. Separated pups did not have access to food or water during their separation period. Litters were always separated and reunited with the mother during the first half of the light phase. The first hour after reuniting the litter with the mother was videotaped. Litters were of different sizes and when possible each litter came from a different breeding pair. A more detailed description of the litters is published elsewhere [19].

#### ‘Endogenous’ model of depression

Flinders Sensitive Lines (FSL) and Flinders Resistant Lines (FRL) rats represent an ‘endogenous’ model of depression [20-23]. Flinders Lines are strains originally obtained by selective breeding of out-bred Sprague-Dawley rats (SD), according to their resistance or sensitivity to anticholinesterase diisopropyl fluorophosphates (DFP) treatment [24]. FSL are congenitally more sensitive to DFP and cholinergic agonists than FRL, which is a neurobiological feature shared with depressed cases in humans [21]. They also show many behavioral similarities to human depressed patients, including decreased psychomotor activity and appetite, cholinergic hypersensitivity, immune and sleep abnormalities including delay in rapid eye movement (REM) sleep but preserved cognitive function and hedonic response [25]. Flinders rats remain a robust model of depression to date [26].

#### mRNA extraction and lab protocols

Mouse brains, livers and spleens were dissected from each animal and frozen on dry ice. Total RNA was extracted from frozen hippocampal tissue and 3-ug RNA was processed using the One Cycle Target Labelling kit (Affymetrix, Santa Clara, CA, USA) and hybridized to the mouse MOE430v2 Gene Expression Array (Affymetrix) following standard Affymetrix protocols. Hippocampal mRNA extraction from Flinders rats was performed by another participating group from the GENDEP project [18,22]. Briefly, cRNA probes were obtained and hybridized to Affymetrix Rat Genome 230 2.0 using Affymetrix’s One-Cycle Eukaryotic Target Labelling Assay protocol. Protocols used for the human post-mortem mRNA extraction are described in detail in the paper by Iwamoto and colleagues [27]. Briefly, total RNA was extracted from 0.1 g of frozen prefrontal cortex tissues using Trizol (Invitrogen, Groningen, The Netherlands). A total of 8 to 10 mg of mRNA was reverse transcribed and synthesized into cDNA, hybridized onto Affymetrix HU95A oligonucleotide arrays and scanned using an HP GeneArray scanner (Hewlett-Packard, Palo Alto, CA, USA). Information on The Stanley Foundation brain collection and Neuropathology Consortium is found elsewhere [28].

#### Human samples

The human samples used in this study were donated to the Stanley Foundation Brain Collection at the Department of Psychiatry, University of the Health Sciences, Bethesda, MD, USA and have been made available to researchers world-wide. Human brain tissues were donated under standardized legislation according to the Uniform Anatomical Gift Act (USA). Information on Stanley Medical Research Institute (SMRI) and its research was offered to the next of kin at the time of the donation. Additional information is publically available from the Stanley Brain Consortium website [29]. The primary transcription-wide analysis was performed and described by Iwamoto and colleagues [27]. For consistency and quality assurance, the same subset has been used without additions or subtractions of cases. All data have been processed from raw files. The samples used consist of post-mortem prefrontal cortex from the Stanley Foundation Neuropathology Consortium from deceased patients affected with major depressive disorder and carefully matched controls. Exclusion criteria include poor mRNA quality and age (>65). A total of 26 samples, 11 cases and 15 controls, were used congruent with the primary data analysis (Table 1). Clinical diagnosis of MDD was made following Diagnostic and Statistical Manual of Mental Disorders – 4^th^ Edition (DSM-IV) diagnostic guidelines and reviewed independently by a pathologist and psychiatrist. Additional information on the human sample can be found in the Iwamoto and colleagues paper [27].

**Table 1:** Genes dysregulated by UCMS

			**Log 2 fold change**				
**Transcript**	**Gene name**	**Pr. Rsum**	**c57**	**DBA**	**FVB**	**129**	**Pfp -Value**
1418687_at	*Arc*	2,841.834	-0.242	-0.279	-0.221	-0.127	<1.00E-04
1452453_a_at	*Camk2a*	2,834.213	0.247	0.276	0.003	0.235	<1.00E-04
1427663_a_at	*Clk4*	4,161.448	-0.420	-0.025	-0.196	-0.289	<1.00E-04
1436983_at	*Crebbp*	1,498.852	0.600	0.156	0.176	0.566	<1.00E-04
1433733_a_at	*Cry1*	5,285.620	0.438	0.113	0.070	0.238	<1.00E-04
1443805_at	*Dact3*	4,920.751	-0.339	-0.097	-0.084	-0.292	<1.00E-04
1438892_at	*Dep1*	3,046.244	0.157	0.142	0.164	0.571	<1.00E-04
1419580_at	*Dlg4*	3,239.403	-0.224	-0.115	-0.177	-0.299	<1.00E-04
1453994_at	*Eml6*	5,673.323	0.164	0.194	0.104	0.189	<1.00E-04
1430436_at	*Fam115a*	4,086.744	0.425	0.025	0.069	0.434	<1.00E-04
1418240_at	*Gbp2*	3,167.576	-0.183	-0.368	-0.335	-0.169	<1.00E-04
1417949_at	*Ilf2*	4,243.511	-0.431	-0.112	-0.115	-0.279	<1.00E-04
1415899_at	*Junb*	3,330.616	-0.246	-0.300	-0.349	-0.232	<1.00E-04
1457899_at	*Kalrn*	4,503.738	0.238	0.169	0.088	0.408	<1.00E-04
1438403_s_at	*Malat1*	1,725.168	-0.183	-0.464	-0.248	-0.139	<1.00E-04
1419568_at	*Mapk1*	923.836	-0.704	-0.328	-0.203	-0.486	<1.00E-04
1420931_at	*Mapk8*	2,498.977	-0.336	-0.180	-0.268	-0.193	<1.00E-04
1425459_at	*Mtmr2*	4,168.453	-0.344	-0.198	-0.206	-0.236	<1.00E-04
1425014_at	*Nr2c2*	3,897.542	0.420	0.016	0.169	0.421	<1.00E-04
1416505_at	*Nr4a1*	4,665.395	-0.234	-0.148	-0.225	-0.161	<1.00E-04
1458176_at	*Per3*	4,870.404	0.269	0.168	0.106	0.222	<1.00E-04
1416211_a_at	*Ptn*	4,211.224	-0.439	-0.151	-0.176	-0.225	<1.00E-04
1440001_at	*Rian*	4,837.821	0.284	0.070	0.192	0.279	<1.00E-04
1439940_at	*Slc1a2*	2,581.498	0.547	0.079	0.059	0.441	<1.00E-04
1444489_at	*Slc25a12*	4,744.455	0.293	0.077	0.117	0.197	<1.00E-04
1421924_at	*Slc2a3*	3,356.425	-0.435	-0.125	-0.202	0.000	<1.00E-04
1421225_a_at	*Slc4a4*	3,960.423	0.408	0.160	0.073	0.278	<1.00E-04
1455876_at	*Slc4a7*	4,614.381	0.313	0.158	0.209	0.235	<1.00E-04
1457357_at	*Tlk2*	3,989.909	0.288	0.100	0.059	0.395	<1.00E-04

Summary of genes found to be differentially expressed in response to the unpredictable chronic mild stress protocol and previously associated with stress response. A stringent cut-off of *P* <1 × 10^-04^ and consistent directionality of fold change was used to identify differentially expressed across all four strains. The table shows the probe set ID, Gene Name, Product Rank Sum (PR.Rsum) value, log 2 Fold change for each or the four strains and *PFP* value.

#### Statistical analysis of microarray data

Probe intensity data from 144 Affymetrix mouse whole-genome oligonucleotide arrays (MOE 430 v2) were normalized and summarized using the Robust Multichip Average (RMA method) [30]. Probe sets that were systematically absent (based on the MAS 5.0 detection present/absent call) across all the arrays were removed leaving 37,231 out of the original 45,101 probe sets. A battery of quality control metrics and exploratory analysis on the 144 arrays identified 10 arrays that differed significantly in quality. These arrays were removed for the purpose of the subsequent analysis; further description on normalization methods is available elsewhere [12,16].

In order to identify genes differentially expressed in response to early and late stress protocols we performed two sets of analyses. First, we compared normalized gene expression measurements between maternally separated animal (MS) and control (CON). Second, we compared normalized gene expression measurements between UCMS and CON. Differences were statistically evaluated using the non-parametric algorithms implemented in the RankProd package in the R environment [31,32]. RankProd enabled us to combine datasets from four different strains using a meta-analysis approach with the RPadvance function. This allowed us to circumvent issues arising from the predominant strain effects by evaluating differences within each strain first. Genes differentially expressed in a single strain were analyzed using rank product (RP) function from the same package, using the ‘data from single origin’ option. The *P*-values were calculated with 1,000,000 permutations, and multiple testing was taken into account by using the percentage of false prediction at the very conservative threshold of *PFP* <0.001. A common method to control for the number of rejected hypothesis in ‘omics’ study is to compute and report the false discovery rate (FDR) as proposed by Benjamini and Hochberg. The RankProd package returns proportion of false positive (PFP), which is a method proposed by Fernando and colleagues. Contrary to FDR, PFP does not rely on the correlation between tests and the number of tests performed [33]. Although PFP and FDR are often equated, the two methods differ in that PFP controls the proportion of accumulated false positives while FDR controls the expected proportion of false positive. FDR is not the best method to use in cases where there is a relationship between variables, which in mRNA studies is generally driven by genetic regulatory pathways and cross hybridization. We therefore corrected using the PFP method across all studies where we use the RankProd algorithim. The genes significantly altered were identified by the PANTHER classification system [34]. Genes with *PFP* <0.001 were subsequently uploaded to the Ingenuity database for pathway analysis with the Ingenuity Pathway Analysis (IPA) software (QIAGEN’s Ingenuity® Pathway Analysis (IPA®, Redwood City, USA) [35].

Expression data from FSL and FRL animals have been made available on the Gene Expression Omnibus (GEO; accession number GS2088, [36]. Data have been processed from raw. CEL files to ensure consistency of data analysis across all animal studies. To control for potential batch effects we combined the rat datasets from two cohorts using the ComBat function built into the inSilicoMerging package for the R environment [37]. Probe sets were normalized and summarized using Robust Multichip Average (RMA method). Probe sets that were systematically absent (based on the MAS 5.0 absent/present detection call) were removed. Probe-set summaries from FSL and FRL were then compared using the RankProd non-parametric algorithm implemented in R using the PRadvance function and single origin option. *P*-values were evaluated using 1,000,000 permutations. A conservative false discovery rate (PFP) threshold of *P* <0.001 and a change fold >1.5 was used. Probe sets that met the statistical thresholds were subsequently annotated using PANTHER [34] to obtain a list of gene symbols. We then matched all genes differentially expressed across all rodent studies using scripts written in Python [38]. Convergent genes differentially expressed across all rodent studies were subsequently analyzed using IPA software. Lastly, all genes differentially expressed in response to both “reactive” and “endogenous” models of depression were used to inform probe set selection in the human study.

Raw scores from 26 Affymetrix human oligonucleotide arrays (HU95A) were normalized and summarized into probe sets using the RMA method, which returned log2 transformed intensities [30]. Intensity distributions, profile correlations and quality control metrics were applied. MAS 5.0 expression values were calculated based on scaling to a target intensity of 100, then transformed by Log2 and calls were computed using the MAS5.0 present/absent algorithm. Affymetrix HU95A incorporates over 12,000 probe sets, tagging the expression of over 5,000 well-characterized genes. Human genes, ortholog to genes differentially expressed across all three rodent studies, were obtained using the Mouse Genome Informatics orthology query [39]. The Affymetrix Netaffx tool [40] was used to identify probe sets on the HU95A chip (Affymetrix) tagging the expression of the human genes. Expression differences between human MDD cases and controls were evaluated using the RankProd non-parametric algorithm implemented in R using the single origin function. Candidate genes in humans informed by the results from the mouse study were considered differentially expressed at a stringent corrected significance threshold *PFP* <0.05 using permutation testing with 1,000,000 permutations.

## Results

### Gene expression profiles in ‘reactive’ depression models

**Figure 2:**
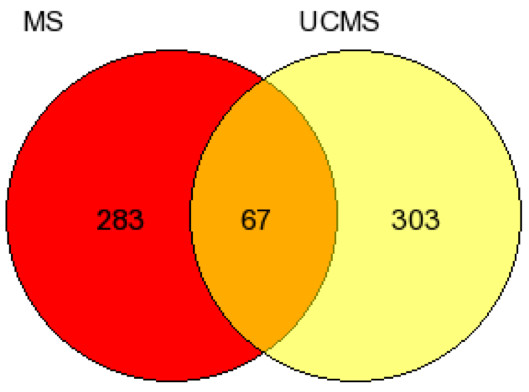
**Venn diagram showing the number of genes significantly altered in response each depressogenic protocol.** A compelling finding is the limited number of overlapping genes (approximately 8.8%) suggesting that etiologically different molecular mechanisms underpin a congruent set of behaviors.

The Rankprod method was used to identify the most robustly differentially expressed genes between ‘late’ (UCMS) stressed animals and control and between ‘early’ (MS) stressed animals and control. We considered only those genes that show consistency in the direction of change across all four strains. Inconsistency in the direction of change indicates Stress x Strain interaction effects, which are not specific to our research question. The results of this analysis uncovered 406 probe sets altered in response to UCMS across all four strains. These probes tag the expression of 370 known genes in mice. A summary of genes uncovered from this analysis with a previous association with stress response or MDD is presented in Table 1. The results reveal a number of genes previously associated with UCMS protocols and believed to play a role in the pathogenesis of MDD. The same analysis was repeated to compare the maternally separated animal (MS) and control. The results from this analysis revealed 396 probe sets differentially regulated in response to the maternal separation protocol. These probe sets could be mapped to 350 known genes in mice. A summary of the top genes differentially expressed in response to maternal separation protocols is presented in Table 2. We then explored the number of altered genes in response to either ‘early’ or ‘late’ stressors as well as the genetic overlap between the two conditions (Figure 2). There were remarkably few. Only 67 genes, less than 8.8% of significantly altered genes were in common between mice exposed to early and late stress paradigms.

**Table 2:** Genes dysregulated by MS

			**Log 2 Fold Change**				
**Transcript**	**Gene Name**	**Pr. Rsum**	**c57**	**DBA**	**FVB**	**129**	**Pfp -Value**
1454655_at	*Dgkd*	3,024.390	0.276	0.399	0.147	0.273	<1.00E-04
1450392_at	*Abca1*	3,716.966	0.014	0.208	0.150	0.319	<1.00E-04
1416250_at	*Btg2*	2,390.318	-0.346	-0.301	-0.161	-0.500	<1.00E-04
1416332_at	*Cirbp*	3,963.875	-0.301	-0.086	-0.237	-0.335	<1.00E-04
1427663_a_at	*Clk4*	3,977.651	-0.349	-0.215	-0.271	-0.127	<1.00E-04
1458518_at	*Cpeb2*	2,642.230	-0.336	-0.401	-0.247	-0.177	<1.00E-04
1451977_at	*Dyrk1a*	4,954.256	-0.133	-0.206	-0.235	-0.168	<1.00E-04
1421142_s_at	*Foxp1*	3,977.517	-0.369	-0.264	-0.153	-0.143	<1.00E-04
1439717_at	*Gabrg3*	5,191.066	-0.174	-0.254	-0.169	-0.021	<1.00E-04
1422223_at	*Grin2b*	4,777.728	-0.220	-0.129	-0.225	-0.223	<1.00E-04
1438441_at	*Id4*	3,829.228	0.313	0.324	0.290	0.046	<1.00E-04
1420931_at	*Mapk8*	2,623.666	-0.158	-0.373	-0.164	-0.333	<1.00E-04
1425459_at	*Mtmr2*	4,660.997	-0.283	-0.212	-0.165	-0.169	<1.00E-04
1437660_at	*Nktr*	5,633.677	-0.089	-0.008	-0.178	-0.226	<1.00E-04
1443970_at	*Ntrk3*	3,929.819	0.261	0.388	0.117	0.254	<1.00E-04
1437213_at	*Nudt21*	4,091.995	0.234	0.294	0.040	0.018	<1.00E-04
1453750_x_at	*Pitpnc1*	5,481.080	-0.305	-0.146	-0.065	-0.179	<1.00E-04
1418015_at	*Pum2*	3,066.939	-0.032	-0.362	-0.146	-0.438	<1.00E-04
1428462_at	*Ppp2r5e*	5,555.808	-0.212	-0.192	-0.085	-0.273	<1.00E-04
1428905_at	*Rraga*	2,709.505	-0.266	-0.447	-0.169	-0.322	<1.00E-04
1421346_a_at	*Slc6a6*	2,560.666	-0.421	-0.418	-0.238	-0.333	<1.00E-04
1420867_at	*Tmed2*	2,172.246	-0.329	-0.291	-0.457	-0.250	<1.00E-04
1435770_at	*Tmx4*	4,098.309	-0.197	-0.078	-0.313	-0.140	<1.00E-04
1459737_s_at	*Ttr*	1,229.204	0.678	0.186	0.074	0.376	<1.00E-04
1420833_at	*Vamp2*	3,820.922	-0.133	-0.172	-0.256	-0.244	<1.00E-04
1450308_a_at	*Xrn1*	2,743.074	-0.175	-0.373	-0.340	-0.306	<1.00E-04
1420816_at	*Ywhag*	1,838.774	-0.265	-0.294	-0.419	-0.362	<1.00E-04
1448219_a_at	*Ywhaz*	3,773.496	-0.314	-0.283	-0.323	-0.252	<1.00E-04

Summary of genes found to be differentially expressed in response to the maternal separation stress protocol. A stringent cut-off of *P* <1 × 10^-04^ and consistent directionality of fold change was used to identify differentially expressed across all four strains. The table shows the probe set ID, Gene Name, Product Rank Sum (PR.Rsum) value, log2 Fold change for each or the four strains and *PFP* value.

The minimal gene expression overlap suggests that the biological mechanisms underpinning ‘reactive’ depression caused by early and late stressors differs considerably. In order to gain further understanding into these differences, genes differentially expressed for each of the two models were analyzed using IPA [35]. This allowed us to uncover gene networks showing the molecular relationship between the genes and evaluate networks according to the fit of significant genes in each dataset [12]. First, we explored gene networks associated with ‘late’ UCMS protocols. A total of 350 genes from our reference list were found on the IPA database. The top two functional networks identified by IPA have a score >42, with 29 reference molecules included in the first network and 23 in the second. Both networks were significantly associated with stress signaling response. The most significant transcriptional regulators included ELK1/2/4 TFIIA, SMARCB, CREB1 and THRB (see Additional file 1: Figure S1 and Additional file 2: Figure S2). We repeated the pathway analysis with genes differentially expressed in response to ‘early’ (MS) stressors. A total of 347 genes from our reference list were found on the IPA knowledge database. IPA returned three networks with a score >40. The associated functions of the top networks include mRNA post-transcriptional modification, protein synthesis and cellular development. The networks are associated with developmental and neurological disorders, which is a good match to the “early” stress protocol used. The top-ranking network (see Additional file 3: Figure S3) includes 29 focus molecules from our reference gene set. The most prominent interacting genes within this network are with the *Yhwaz* and *Yhwag*. These genes are of particular interest as they have been systematically uncovered across several proteomic and transcriptomic studies from the GENDEP project in both mice and rats [12,13,17,22]. These genes show a direct interaction with the STK25 kinase, which plays a role in stress response. The second network (Additional file 4: Figure S4) is composed of 27 molecules from our reference dataset. This network is centered on the NF-κB complex. The nuclear factor-κB (NF-κB) is a ubiquitous transcription factor involved in the regulation of gene expression and cell stress response and cell proliferation. Interestingly, NF-κB can be activated by different stimuli, including cytokines (such as TNF-α and IL-1): this finding is congruent with the inflammation hypothesis for MDD [41-45].

### Gene expression profiles in ‘endogenous’ depression models

**Figure 3:**
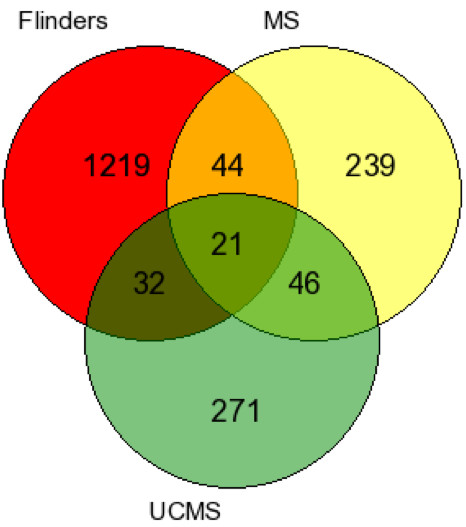
**Venn diagram showing the number of genes overlapping across all rodent studies.** Only 67 genes were differentially regulated in response to both early (MS) and late stressors (UCMS) pointing at minimal genetic overlap. However, many of these genes (approximately 30%) were also differentially regulated in an endogenous rat model of depression. The replication of these genes in a different organism that shows congenital depression-like symptoms, points at molecular mechanisms that may be involved in the human pathology.

**Figure 4:**
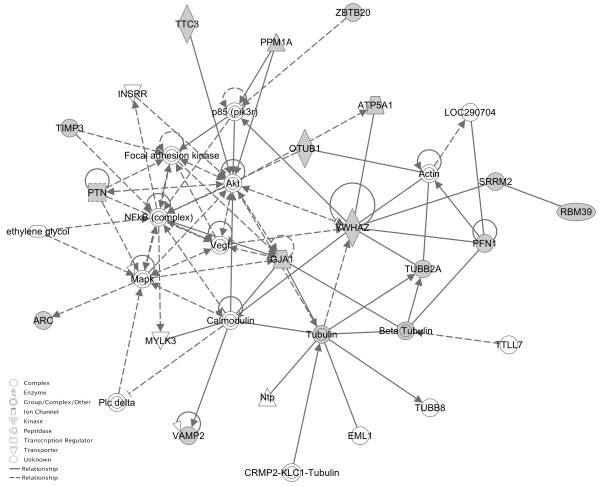
**Network analysis performed on converging genes differentially regulated in response to different stresses in mouse and between different selected Flinders lines.** The pathway comprises over half the reference molecules uploaded to the Ingenuity database system (12 out of the 21 reference molecules). The pathway implicates a number of genes previously associated with MDD and antidepressant treatment response, including *Ppm1a, Ywhaz, NkFb* and *Mapk.*

To gain an understanding of the similarities between stress-induced ‘reactive’ depression and a congenital ‘endogenous’ model of depression, we compared genes differentially regulated in response to early (MS) and late stress (UCMS) with mRNA differences between Flinders sensitive and resistant lines. Flinders lines are a genetic animal model of depression that allows us to cross-validate stress-altered genes within a parallel, independent mRNA study where depressive-behaviors occur in the absence of environmental stressors. The RankProd algorithm and conservative cut-offs described previously was used to evaluated mRNA differences between Flinders Sensitive and Flinders Resistant lines. The results revealed 715 down-regulated and 1,145 up-regulated probe sets. To obtain a list of gene names, probe sets obtained were subsequently annotated using PANTHER [34]. The probe sets tagged the expression of 501 down-regulated and 727 up-regulated genes. A total of 1,228 genes were used for cross-validation with the mouse study. First, we explored the genomic overlap between maternally deprived animals and Flinders line rats. From a total of 350 genes differentially regulated in maternally deprived mice, a total of 65 genes (19%) were also differentially regulated in rats. The same comparison was performed with genes differentially expressed in mice exposed to UCMS. A total of 52 genes (11%) were differentially expressed between ‘late’ stress animals and Flinders rats. A compelling finding is that 21 genes are differentially expressed in rats and in both early and late stressed mice (Figure 3). This is an important genetic overlap given that only 67 genes were commonly expressed between early and late stressed animals in the first place. Validations in an independent, methodologically different study using a genetic model of depression point to an important genetic overlap between stress-related and syndrome-related mechanisms. In order to gain further biological insight, genes significantly altered in response to both stresses in mice and between Flinders Sensitive and Resistant lines were carried forward for analysis using Ingenuity’s IPA system. All 21 genes were found in the Ingenuity reference database. A significant network with a score >40 consisting of over 55% of the reference molecules (12/21) was revealed (Figure 4). Among the genes in the pathway, four genes (*Ywhaz*, *Ppm1a*, N*kfb* and *Mapk1*) are of particular interest as they have been previously reported across different “omics” GENDEP investigations [12,17,46-48].

### Translating findings to humans

From a total of 21 genes differentially expressed across all three rodent studies, 15 human orthologs were found. The expression of these 15 genes is tagged by 21 probe sets on the Affymetrix HU95A oligonucleotide array. The RankProd algorithm was used to evaluate expression between post-mortem cases and controls. Out of a total of 15 genes, the *VAMP-2* is significantly down-regulated after correcting for the number of multiple non-independent tests using a prediction of false discovery rate of *PFP* <0.05 (Table 3). In our study, the Vesicle-Associated Membrane Protein 2 (*VAMP-2: Synaptobrevin2)* gene is significantly altered across all rodent studies and in the human study.

**Table 3:** Convergent genes across all rodent studies

**Probe Set ID**	**Gene symbol**	**RP/Rsum**	***PFP***	***P*-value**
1034_at	*TIMP3*	10.3564	1.0512	0.6398
1035_g_at	*TIMP3*	10.788	0.9069	0.7097
1235_at	*YWHAZ*	8.1361	1.8677	0.2436
2018_at	*GJA1*	10.6102	0.9224	0.6818
296_at	*TUBB2A*	8.1718	1.4332	0.2493
297_g_at	*TUBB2A*	10.38	0.9872	0.6438
32254_at	*VAMP2*	9.7002	0.0024	0.5228
32531_at	*GJA1*	5.6354	0.4128	0.018
32572_at	*USP9X*	9.6037	1.0556	0.5049
32761_at	*SRRM2*	11.6727	0.9074	0.8285
34387_at	*LPGAT1*	10.5963	0.9769	0.6796
34642_at	*YWHAZ*	6.7492	0.8804	0.0766
36307_at	*ARC*	8.5526	1.0273	0.3127
36760_at	*YWHAZ*	9.8422	0.9712	0.5489
38211_at	*ZBTB20*	8.6008	0.8204	0.321
38710_at	*OTUB1*	9.566	1.145	0.4978
39331_at	*TUBB2A*	8.2579	1.2097	0.263
39725_at	*RBM39*	10.946	0.888	0.7336
40096_at	*ATP5A1*	8.5727	0.9088	0.3161
40125_at	*CANX*	8.2587	1.0086	0.2631
968_i_at	*USP9X*	11.0827	0.8662	0.7532

The expression profile of these genes is tagged by 21 probe sets. Expression differences between cases and control were evaluated using the RankProd algorithm using probability of false positive (*PFP*) of less the 0.05 to control for the number of multiple testing. The table shows Probe Set ID, Gene Symbol, RankProd value, corrected *PFP* value and uncorrected -value. The only gene that survives correction for multiple testing in the human analysis is the *VAMP-2* gene.

## Discussion

The main objective of this study was to compare the genomic signatures of ‘reactive’ and ‘endogenous’ models of depression in three rodent studies and translate these findings in a human study. We found that all three animal models of depression had largely unique gene-expression profiles indicating divergent molecular mechanisms. Nevertheless, a small set of genes was consistently dysregulated across each paradigm and in the post-mortem brain tissue of depressed patients, which may represent a final common pathway to the disorder.

### Gene-expression profiles of ‘endogenous’ and ‘reactive’ models of depression

Consistent with our hypothesis and with previous findings, the gene-expression profiles of both of our ‘reactive’ models of depression were largely distinct from our ‘endogenous’ model. Interestingly, this differed according to which ‘reactive’ depression paradigm was compared. For the early or stress ‘reactive’ model 19% of genes overlapped with the endogenous model, while for the late stress ‘reactive’ model the overlap was considerably lower at just 11%.

Surprisingly, the genomic signatures of our two ‘reactive’ models were more distinct from one another than the ‘endogenous’ model with fewer than 9% of genes shared between the two paradigms. This suggests that the two different models result in depressive-like behavior in mice through distinct biological mechanisms. Interestingly, gene pathway analysis returned plausible functional networks, with the more significant network for ‘early’ stressed animals associated with neurodevelopmental disorders and those of ‘late’ stressed animals associated with cell stress response and cell-signaling. Taken together, our results suggest that early exposure to stress modulates the expression of genes belonging to pathways associated with neurodevelopmental mechanisms. These changes may condition an individual’s exposure to later stresses and response to pharmacological and behavioral interventions later in life in yet unclear ways. Conversely, late onset stresses may act primarily on brain neurochemistry with neurostructural changes occurring via the cascading effects of neurochemically-related mechanisms, including neurogenesis and apoptosis [49].

### Genes differentially regulated across all three paradigms

While each of our three animal models of depression showed largely distinct gene-expression profiles, a set of genes were differentially regulated across all three paradigms. Pathway analysis of these genes revealed a gene network which included *Ppm1a*, *Ywhaz, Nkfb* and *Mapk*.

All four genes have each been implicated in both the etiology of MDD and the response to treatment and may, therefore, represent a final common pathway to the disorder*.* We previously reported that the expression *ppm1a* was significantly modulated by the antidepressant nortriptyline. We have also shown that several single nucleotide polymorphisms in the human ortholog of this gene (*PPM1A*) predict a response to the same drug in a parallel human pharmacogenetic study [46]. Tyrosine 3-monooxygenase/tryptophan 5-monooxygenase activation protein (*Ywhaz*) has systematically been uncovered across several GENDEP studies and plays a role in cell proliferation and neurogenesis, which is a current explanatory model of MDD [50-54]. Moreover, it interacts with IRS1 protein and the MAPK pathway by modulating the activation of JNK1 and p38 MAPK both of which have been systematically associated with depressive mechanisms [52-56]. Lastly, the *Nfkb* gene has extensively been associated with peripheral inflammation and is consistent with the inflammation hypothesis for MDD [57].

#### Convergent animal-human genes

Animal models are an attractive proposition for the study of mood disorders as they allow access to disease relevant brain tissues and to control for environmental conditions. However, given the nature and characteristic of psychiatric disorders, there are aspects of these illnesses that can only be studied in humans. We therefore attempted to translate our set of convergent genes emerging from the rodent studies in a matching human post-mortem mRNA study of depressed cases and controls. One gene, the *VAMP-2* gene, remained significantly down-regulated after correcting for multiple testing in the human study. The vesicle-associated membrane protein (*VAMP-2*; synaptobrevin2) plays a role in the molecular regulation of transmitter release at the presynaptic plasma membrane. The expression of *VAMP-2* has been found to be altered in both schizophrenia and bipolar disorder within a combined microarray analysis of the Stanley Foundation's brain collections [58]. Moreover, several other studies have implicated this gene in Axis-I psychiatric disorders and in antidepressant treatment response [59-62]. Previous studies have also shown that the *VAMP2*/synaptobrevin-2 gene is increased in rat frontal cortex after chronic antidepressant treatment and repeated electro-convulsive therapy (ECT), although the finding has not been consistently replicated [63,64].

#### Implications

If replicated, the results of our study may have far reaching implications for both personalized medicine for MDD and case-control studies of the disorder.

Our findings suggest that etiological factors (such as proximal and distal stressors) could be used to indicate the molecular mechanisms at work in a given patient and, therefore, select the most effective treatment. Indeed, several studies have shown that proximal and distal stress predicts a response to antidepressants. Interestingly, while proximal stressors, such as divorce or job loss, have been linked with a good response [3], distal stressors, such as childhood maltreatment, are associated with a less favorable outcome [5]. Our results suggest that these contradictory findings may be explained by the divergent molecular mechanisms underlying ‘reactive’ depression caused by early versus late stressors. Nevertheless, further studies in clinical samples would be required to test this hypothesis.

Heterogeneity in the molecular mechanisms underlying depression could explain why, despite considerable efforts, genome-wide association studies (GWAS) of depression have yet to identify statistically significant associations with MDD [65]. This same heterogeneity may also explain the paucity of findings from pharmacogenetic studies of MDD, including the very large GWAS, NEWMEDS [66]. If the molecular mechanisms underlying MDD differ according to stress, it is plausible that different genetic variants would predict response to treatment in stressed and non-stressed individuals. In line with this, several studies have shown that genetic variants and stress have interdependent effects on antidepressant response [67].

While our findings highlight the heterogeneity of depression, they also suggest that a small set of genes may be involved in a final common pathway to the disorder. Replication of these findings in further transcriptomic studies of clinical samples is necessary before any firm conclusions can be drawn about the role of these genes in MDD. However, if they are successful, the existence of a final common pathway provides an exciting prospect for the development of novel antidepressants. If indeed the heterogeneity of MDD explains inter-individual variation in treatment response, it is plausible that antidepressants, which target this final common pathway, may prove to be effective for all patients, regardless of their etiological factors.

#### Limitations

Our findings should be considered in the context of several important limitations.

First, we used whole genome gene-expression data from four different samples in our study. While this approach allowed us to conduct integrative analyses across species and translate our findings from rodents to humans, it also meant that our analyses were subject to multiple testings. We used stringent thresholds both within and across analyses in order to protect against the risk of false positive findings. Nevertheless, in taking such an approach it is possible that we inflated the number of false negatives. Further replication of our results in larger independent samples is therefore necessary to confirm our findings.

Second, our rodent models focused exclusively on gene-expression in the hippocampus and did not include further brain structures implicated in the neurobiology of MDD, such as the amygdala. Moreover, limited public human depression hippocampal transcript array data were available at the time of analysis which meant that our human study used gene-expression data collected from a different, but still disease relevant, brain region (the prefrontal cortex). It is plausible, therefore, that the use of a different brain region resulted in false negatives in the human component of our study. Our findings, therefore, require confirmation in further brain regions in both animal and human samples.

Finally, it is important to note that rodents do not capture the complex characteristics of psychiatric illnesses that can only be fully investigated in human studies. Nevertheless, there are many advantages of animal models, which allow access to disease relevant brain tissues and control of environmental conditions. In the current study, we attempted to capture the full potential of both animal and human studies by conducting integrative analyses using several independent animal studies and translating results in a disease-relevant but different brain region in humans.

## Conclusion

It is largely accepted that there are multiple causal pathways to MDD consisting of different combinations of genetic and environmental risk factors [67]. However, it remains unclear whether these factors converge on a unitary molecular mechanism underlying MDD or MDD consists of a heterogeneous group of disorders with multiple causal factors and distinct molecular mechanisms. Our findings provide support for both of these hypotheses. Using an animal model, we have shown that the presence and timing of stress determines distinct molecular processes underlying depressive behavior. However, we also identified a small set of genes which were consistently dysregulated across each stress paradigm and in post-mortem brain tissue of depressed patients suggestive of a final common pathway to the disorder. These genes included *VAMP-2,* a gene which has previously been associated with Axis-I disorders including MDD, bipolar depression, schizophrenia and with antidepressant treatment response.

Careful consideration of the etiological pathways to MDD may be key to dissecting the heterogeneity of the disorder and understanding and predicting response to treatment. Nevertheless, a final common pathway which unites the disparate etiologies of MDD may yet provide a target for novel treatments which are effective for all, rather than just subsets of patients.

## Abbreviations

CON: Control; DFP: Diisopropyl fluorophosphates; FDR: False discovery rate; FRL: Flinders Resistant Line; FSL: Flinders Sensitive Line; GWAS: Genome Wide Association Study; HIP: Hippocampus; IPA: Ingenuity Pathway Analysis; MDD: Major Depressive Disorder; MS: Maternal Separation; PFP: Proportion of false positive; PND: Post Natal Day; RMA: Robust Multichip Averaging; RP: Rank Prod; SSRIs: Selective Serotonin Reuptake Inhibitors; TCAs: Tricyclic Antidepressants; UCMS: Unpredictable Chronic Mild Stress.

## Competing interests

Prof. Peter McGuffin, Dr. Enrico Domenici and Dr. Lucia Carboni have received consultancy fees and honoraria for participating in expert panels from pharmaceutical companies, including Roche and GlaxoSmithKline. All other authors report no biomedical financial interests or potential conflicts of interest.

## Authors’ contributions

KM and RK analyzed the data and conceived the study. MGT and AL contributed to the analyses and methods section. ED and LC conducted the study on the Flinders rats and contributed to the revisions of the manuscript. RU contributed to the design. PM is the GENDEP PI and contributed to the revision and LS conducted the mouse study and revised the manuscript. All authors read and approved the final version of the manuscript.

## Pre-publication history

The pre-publication history for this paper can be accessed here:

http://www.biomedcentral.com/1741-7015/12/73/prepub

## Supplementary Material

Additional file 1: Figure S1 Gene network obtained from genes differentially expressed in response to the UCMS protocol. The most significant network returned from the Ingenuity Pathway Analysis software for genes differentially expressed in response to Unpredictable Chronic Mild Stress. The network consists of 29 reference molecules and is significantly associated with cell stress response. Click here for file

Additional file 2: Figure S2 Second gene network obtained from genes differentially expressed in response to the UCMS protocol. A second significant network with a score >42 returned by the Ingenuity Pathway Analysis software for genes differentially expressed in the mouse study in response to the Unpredictable Chronic Mild Stress protocol. The pathway includes 23 reference molecules and it is also associated with cell stress response. The pathway is centered on the ELK complex hub and is of particular interest as it shows the VAMP-2 complex and its association with the N-type calcium channel and potassium channel. Click here for file

Additional file 3: Figure S3 Gene network obtained from genes differentially expressed in response to the Maternal Separation protocol. The most significant network returned from the Ingenuity Pathway Analysis software for genes differentially expressed in response to the maternal separation depressogenic protocol. Of particular interest is the presence of the *Yhwaz* reference molecule. The *Yhwaz* gene has been systematically uncovered across several animal studies and been shown to influence neurotransmission of dopamine by regulating exocytosis or phosphorylation of synaptic proteins. The pathway consists of 29 reference molecules and is associated with cell stress response. Click here for file

Additional file 4: Figure S4 Second Gene network obtained from genes differentially expressed in response to the Maternal Separation protocol. Second pathway returned from the Ingenuity Pathway Analysis software for genes differentially expressed in response to the maternal separation protocol in mouse. This pathway includes 27 reference molecules and is centered on the NF-κB hub. The pathway is associated with cell proliferation and the NF-κB hub has been previously found to be associated with inflammation. Activation of the NF-κB transcription family, by nuclear translocation of cytoplasmic complexes, plays a central role in inflammation [68]. Human studies have shown that MDD patients with increased early life stress exhibit enhanced inflammatory responsiveness to psychosocial stress [69]. Click here for file
