# Supplementary figures and images for: The endogenous and reactive depression subtypes revisited: integrative animal and human studies implicate multiple distinct molecular mechanisms underlying major depressive disorder
Source: BMC Med. 2014 May 7;12:73. doi: 10.1186/1741-7015-12-73 (PMC4046519; doi:10.1186/1741-7015-12-73)

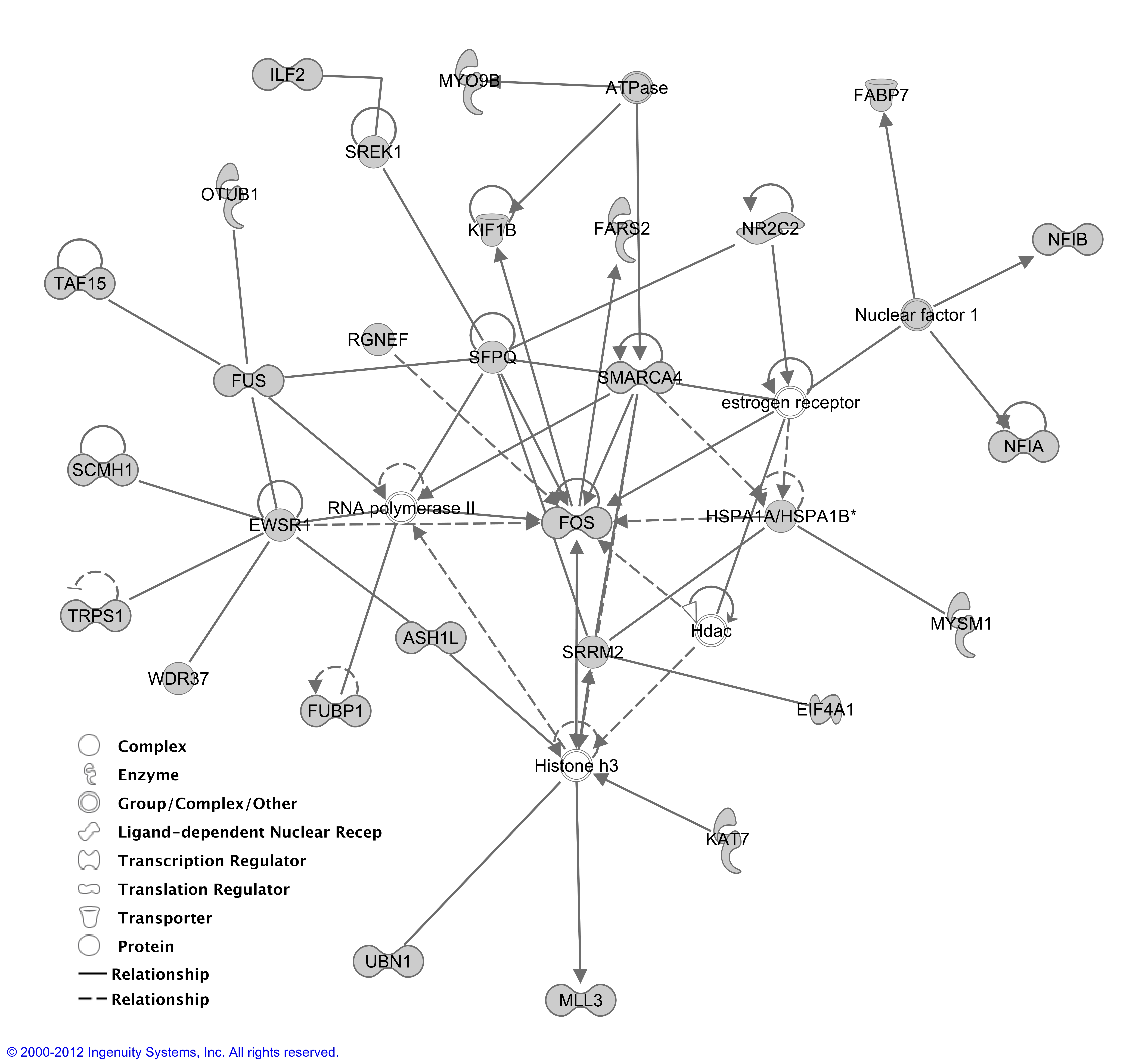

Supplement: Additional file 1: Figure S1 — Gene network obtained from genes differentially expressed in response to the UCMS protocol. The most significant network returned from the Ingenuity Pathway Analysis software for genes differentially expressed in response to Unpredictable Chronic Mild Stress. The network consists of 29 reference molecules and is significantly associated with cell stress response. [file 1741-7015-12-73-S1.png]

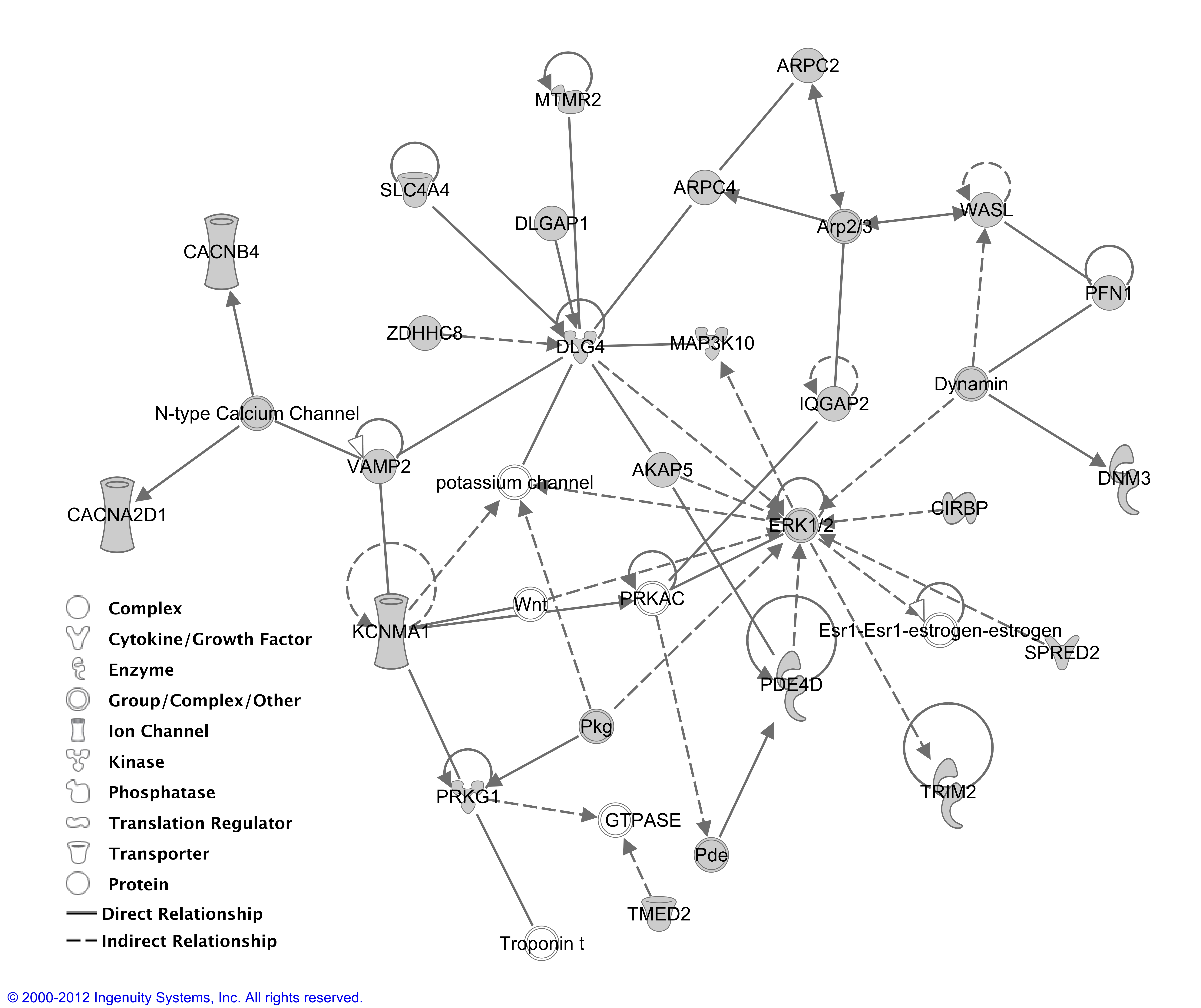

Supplement: Additional file 2: Figure S2 — Second gene network obtained from genes differentially expressed in response to the UCMS protocol. A second significant network with a score >42 returned by the Ingenuity Pathway Analysis software for genes differentially expressed in the mouse study in response to the Unpredictable Chronic Mild Stress protocol. The pathway includes 23 reference molecules and it is also associated with cell stress response. The pathway is centered on the ELK complex hub and is of particular interest as it shows the VAMP-2 complex and its association with the N-type calcium channel and potassium channel. [file 1741-7015-12-73-S2.png]

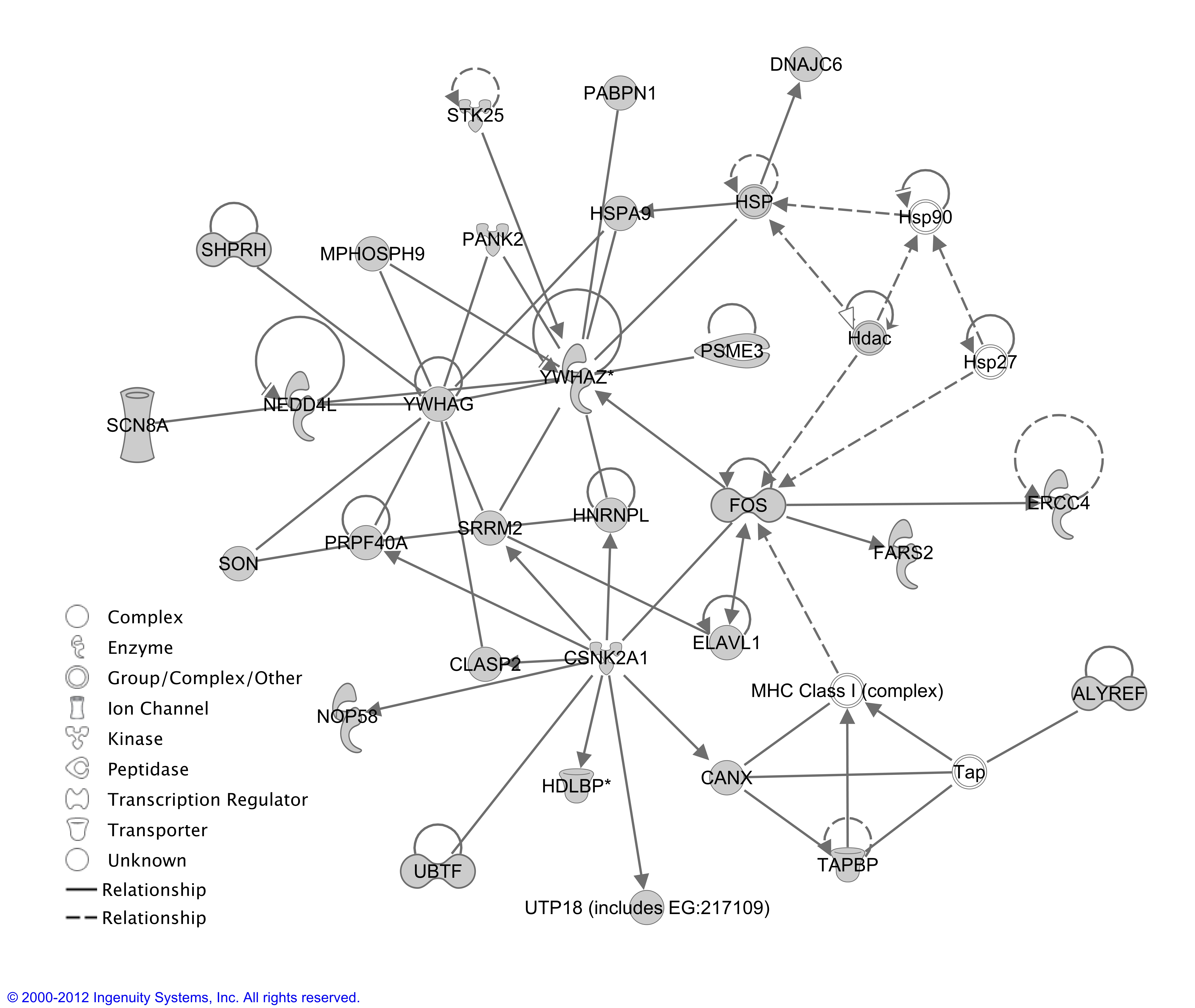

Supplement: Additional file 3: Figure S3 — Gene network obtained from genes differentially expressed in response to the Maternal Separation protocol. The most significant network returned from the Ingenuity Pathway Analysis software for genes differentially expressed in response to the maternal separation depressogenic protocol. Of particular interest is the presence of the Yhwaz reference molecule. The Yhwaz gene has been systematically uncovered across several animal studies and been shown to influence neurotransmission of dopamine by regulating exocytosis or phosphorylation of synaptic proteins. The pathway consists of 29 reference molecules and is associated with cell stress response. [file 1741-7015-12-73-S3.png]

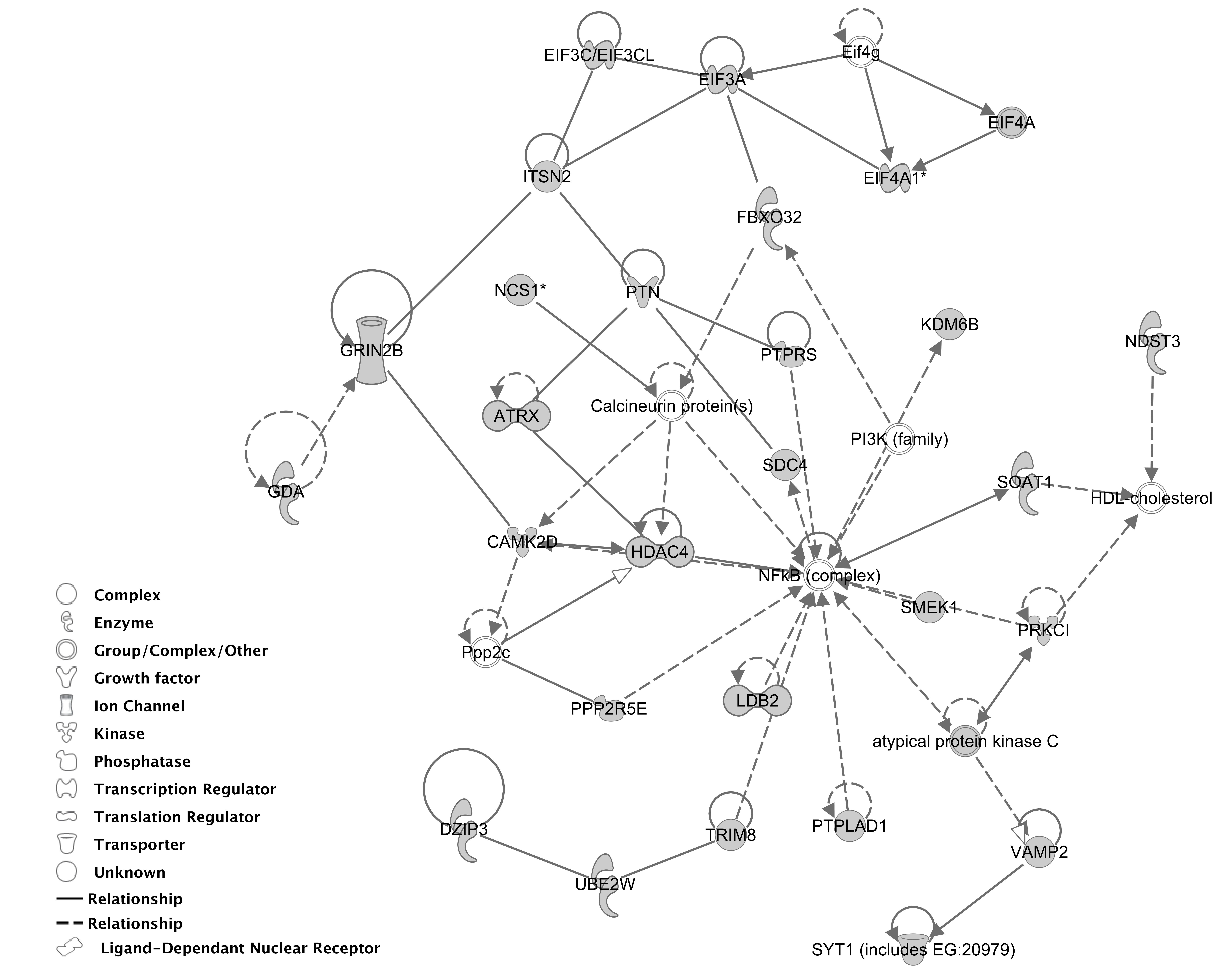

Supplement: Additional file 4: Figure S4 — Second Gene network obtained from genes differentially expressed in response to the Maternal Separation protocol. Second pathway returned from the Ingenuity Pathway Analysis software for genes differentially expressed in response to the maternal separation protocol in mouse. This pathway includes 27 reference molecules and is centered on the NF-κB hub. The pathway is associated with cell proliferation and the NF-κB hub has been previously found to be associated with inflammation. Activation of the NF-κB transcription family, by nuclear translocation of cytoplasmic complexes, plays a central role in inflammation [68]. Human studies have shown that MDD patients with increased early life stress exhibit enhanced inflammatory responsiveness to psychosocial stress [69]. [file 1741-7015-12-73-S4.png]
